# Integrative Functional Genomics of Hepatitis C Virus Infection Identifies Host Dependencies in Complete Viral Replication Cycle

**DOI:** 10.1371/journal.ppat.1004163

**Published:** 2014-05-22

**Authors:** Qisheng Li, Yong-Yuan Zhang, Stephan Chiu, Zongyi Hu, Keng-Hsin Lan, Helen Cha, Catherine Sodroski, Fang Zhang, Ching-Sheng Hsu, Emmanuel Thomas, T. Jake Liang

**Affiliations:** Liver Diseases Branch, National Institute of Diabetes and Digestive and Kidney Diseases, National Institutes of Health, Bethesda, Maryland, United States of America; University of Alabama at Birmingham, United States of America

## Abstract

Recent functional genomics studies including genome-wide small interfering RNA (siRNA) screens demonstrated that hepatitis C virus (HCV) exploits an extensive network of host factors for productive infection and propagation. How these co-opted host functions interact with various steps of HCV replication cycle and exert pro- or antiviral effects on HCV infection remains largely undefined. Here we present an unbiased and systematic strategy to functionally interrogate HCV host dependencies uncovered from our previous infectious HCV (HCVcc) siRNA screen. Applying functional genomics approaches and various *in vitro* HCV model systems, including HCV pseudoparticles (HCVpp), single-cycle infectious particles (HCVsc), subgenomic replicons, and HCV cell culture systems (HCVcc), we identified and characterized novel host factors or pathways required for each individual step of the HCV replication cycle. Particularly, we uncovered multiple HCV entry factors, including E-cadherin, choline kinase α, NADPH oxidase CYBA, Rho GTPase RAC1 and SMAD family member 6. We also demonstrated that guanine nucleotide binding protein GNB2L1, E2 ubiquitin-conjugating enzyme UBE2J1, and 39 other host factors are required for HCV RNA replication, while the deubiquitinating enzyme USP11 and multiple other cellular genes are specifically involved in HCV IRES-mediated translation. Families of antiviral factors that target HCV replication or translation were also identified. In addition, various virologic assays validated that 66 host factors are involved in HCV assembly or secretion. These genes included insulin-degrading enzyme (IDE), a proviral factor, and N-Myc down regulated Gene 1 (NDRG1), an antiviral factor. Bioinformatics meta-analyses of our results integrated with literature mining of previously published HCV host factors allows the construction of an extensive roadmap of cellular networks and pathways involved in the complete HCV replication cycle. This comprehensive study of HCV host dependencies yields novel insights into viral infection, pathogenesis and potential therapeutic targets.

## Author Summary

Hepatitis C virus (HCV) is a positive strand RNA virus that belongs to the *Flaviridae* family. Chronic HCV infection is a leading cause of end-stage liver disease, which is associated with significant morbidity and mortality in the world. Our recent genome-wide siRNA screen has revealed that HCV depends extensively on host factors for efficient infection and propagation. Here we systematically and functionally catalogued these host dependencies to various stages of the HCV replication cycle. Applying systems virology and functional genomics approaches with various *in vitro* HCV model systems, we further defined multiple previously unrecognized host factors or pathways that are involved in either HCV entry, IRES-mediated translation, RNA replication, or assembly/secretion. By bioinformatics meta-analyses and literature mining of existing publications and databases, we constructed an extensive roadmap of the cellular networks and pathways requisite for the complete HCV replication cycle. Our study yields novel insights into viral infection, pathogenesis and potential therapeutic targets. Furthermore, this study serves as a valuable reference source for subsequent work on host pathways and virus-host interactions in general.

## Introduction

Hepatitis C virus (HCV) is a hepatotropic member of the *Flaviridae* family and a primary etiologic agent of chronic hepatitis that can progress to cirrhosis and hepatocellular carcinoma (HCC) [1]. Until recently, standard therapy for hepatitis C was a combination of peginterferon and ribavirin, curing only about half of the patients with substantial side effects [2]. The recent development of direct-acting antivirals (DAAs) significantly improves treatment response in patients infected with HCV genotype 1 [2]. However, the newer regimens are still suboptimal and problematic concerning adverse effects, viral resistance, drug-drug interactions and variable efficacies among HCV genotypes [2]. Consequently, there is a need for developing innovative treatment options for difficult-to-treat patients. HCV exploits host factors extensively for infection and propagation [3], [4], [5], [6]. Identification of these host dependencies may provide not only potential antiviral targets, but also critical insights into mechanisms of HCV-mediated pathogenesis and chronic liver disease.

The replication cycle of HCV broadly encompasses viral entry, viral genome translation and replication, and virion assembly and secretion [7]. The virus enters hepatocytes through several previously identified cell surface molecules and other yet-undefined host factors [8]. Clathrin-mediated endocytosis and fusion leads to uncoating and cytosolic release of viral genome, which then traffics to rough endoplasmic reticulum (ER), where viral polyproteins are translated via an HCV IRES-mediated mechanism. Host and viral encoded proteases process the viral polyprotein into structural and nonstructural proteins [7]. HCV genomic replication occurs in a membranous web structure derived from the ER [7]. Progeny viral genomes are translocated to the surface of lipid droplets (LDs) or LD-associated ER membrane, and assembled into virions, which then complex with lipoproteins to egress the cell [9]. Although significant progress has been made in identifying host factors for HCV propagation, the complex interactions between the entire HCV replication cycle and cells and the underlining mechanisms of actions remain elusive.

Cell-based genetic screening combined with bioinformatics analyses has proved to be highly effective approaches for providing broad roadmaps of host-virus interactions and viral pathogenesis [10], [11], [12]. In a recent effort to define host factors associated with productive HCV infection, we performed a genome-wide small interfering RNA (siRNA) screen, using an infectious HCV cell culture (HCVcc) system [4]. The screening strategy was refined to identify host dependencies in the entire HCV life cycle. With a stringent selection criterion, we uncovered and subsequently validated 237 host proviral factors (HPFs) and 25 host antiviral factors (HAFs) [4]. Moreover, other genome-wide or targeted siRNA screens using various HCV assays also provided additional source of information on cellular factors involved in HCV infection and propagation [5], [6], [13], [14], [15].

Genes identified from the siRNA screens represent a starting point for defining the comprehensive interactions between HCV and cell. How the co-opted host functions are involved in individual steps of HCV replication cycle remains to be elucidated. Further efforts exploring the functions of these cellular genes in HCV pathogenesis are also needed. In this study we applied a systematic strategy to functionally investigate HCV host dependencies uncovered from our genome-wide siRNA screen. We generate an extensive list of host factors and signaling pathways involved in various stages of HCV replication cycle and hence reconstruct a global roadmap of cellular interactive and regulatory networks in HCV infection.

## Results

### Characterization of host dependencies in complete HCV replication cycle

**Figure 1:**
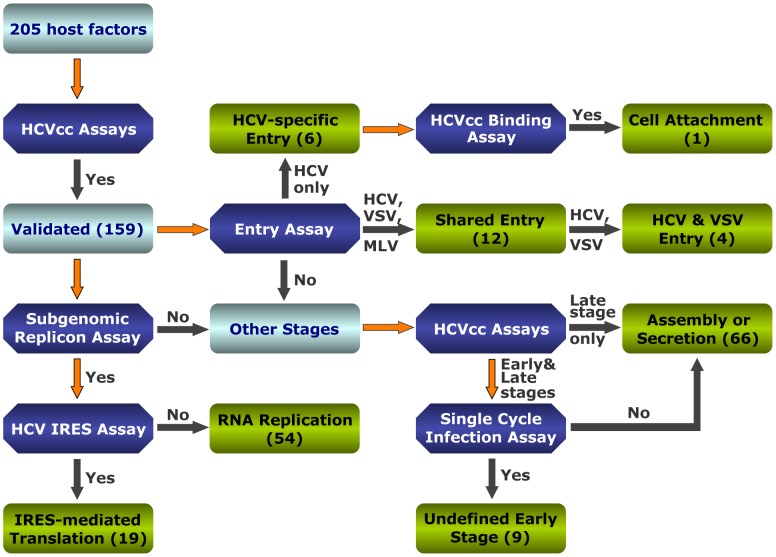
Strategies to interrogate host dependencies involved in the complete HCV replication cycle. Flow diagram of various virologic studies for identification of host factors involved in various steps of the HCV replication cycle. Various virologic assays and the number of validated genes at each stage are indicated. Forty-six genes were not validated by virologic assays of cells treated with OTP SMARTpool siRNAs (either negative results or inconsistent border-line positive results) and therefore were considered as false-positives.

HCV infection consists of multiple distinct and sequential steps, from entry to assembly and secretion. Various *in vitro* models have been developed to study specific aspects of viral infection and interactions of HCV with its host (Illustrated in Figures 1 and S1). These include HCV pseudoparticles (HCVpp) to study viral entry [16], HCV subgenomic replicon to study viral RNA replication [17], HCV IRES-driven construct to examine viral protein translation [18], and HCVcc system that recapitulates the entire HCV life cycle [19], [20], [21]. In addition, a *trans*-packaging system that generates single-round infectious HCV (HCVsc) has been established to distinguish between early stages of entry to replication and late stages of assembly to secretion [22], [23].

To functionally interrogate HCV host dependencies uncovered from the genome-wide siRNA screen [4] and study how these cellular functions are relevant for the HCV replication cycle, we selected 205 host factors of interest based on their known molecular functions and potential interactions with HCV. These host factors include 181 genes identified from the original siRNA screen [4]. Genes from the siRNA database that were not studied further here are either known HCV interacting factors or those with little known functions. Twenty-four additional cellular genes that did not score positive in the siRNA screen but appeared functionally relevant in subsequent bioinformatic analysis were also studied (Table S1). This set of genes might represent false negatives of the RNAi screen – a major caveat that stems from inefficient targeting, nonspecific toxicity or other unappreciated circumstances [24], [25]. We applied a different set of siRNAs, the Dharmacon 2^nd^ generation ON-TARGETplus (OTP) siRNA library to target these genes. The original siRNA screen was conducted with the siGENOME SMARTpools [4]. The newer OTP pools are designed to reduce off-target effects while maintaining siRNA potency. All 205 host genes were subjected to various virologic assays with the above-mentioned *in vitro* HCV assays (Figure S1). A thorough evaluation of the data and reconciliation of minor discrepancies designates these host factors to various stages of HCV replication cycle (Figure 1 and Tables S2, S3, S4, S5, S6, S7, S8).

### Identification of novel HCV entry factors

HCV entry relies on complex interactions between the virus and host cell [8]. Despite multiple host factors and cellular machineries have been implicated in viral entry, their mechanisms of actions remain obscure. Moreover, additional cellular co-factors and signaling pathways important for HCV entry are yet to be defined.

**Figure 2:**
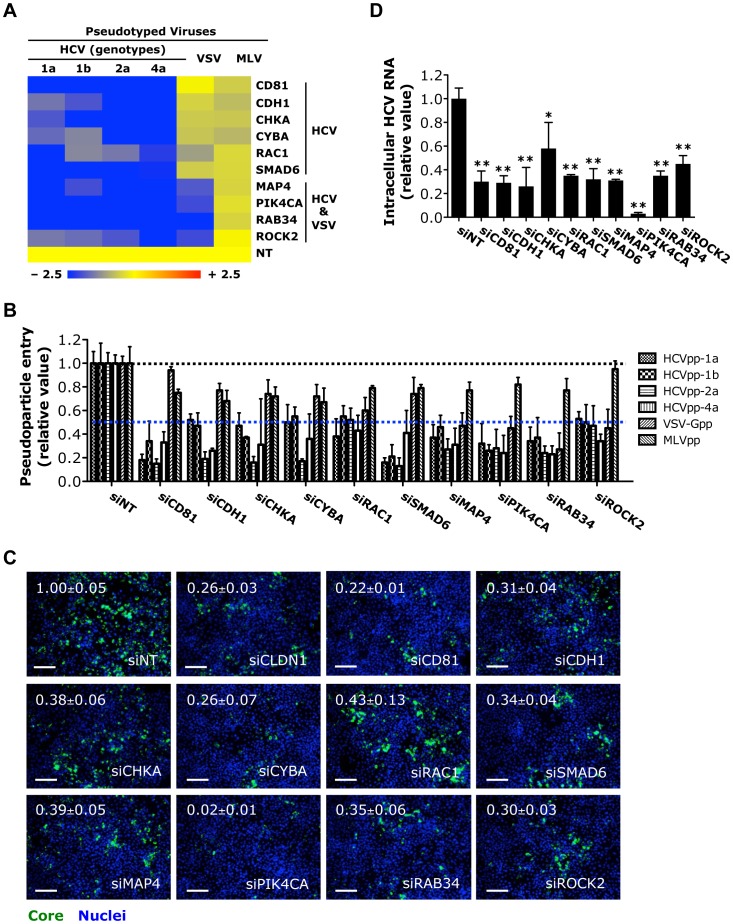
Identification of HCV entry factors. A) The effects of siRNA-mediated gene silencing on infection of firefly luciferase-encoded pseudotyped viruses bearing HCV, VSV or MLV envelopes in Huh7.5.1 cells. Inhibition in the heatmap is shown as blue (strong inhibition) to yellow (little or no inhibition), comparing with non-targeting control (NT). B) Firefly luciferase assay that reflects HCVpp (various genotypes), VSV-Gpp or MLVpp entry of Huh7.5.1 cells treated with siRNAs targeting various indicated viral entry factors. Data shown in A and B are subsets of those in Table S4. C) Image illustration and quantitative analyses of HCV core staining in Huh7.5.1 cells depleted of various viral entry factors by siRNAs and subsequently infected with HCV at an M.O.I. of 0.5 for 48 h. Percentages of core positive cells were quantified and normalized to siNT control (set as 1). Green: HCV core, blue: nuclei. Magnification 20×. Scale bars represent 100 µm. D) RT-PCR quantification of intracellular HCV RNA levels in Huh7.5.1 cells treated with indicated siRNAs prior to infection with HCV. Cells were harvested at 48 h post-infection, and total cellular RNA was then extracted. All values were normalized as relative to siNT (non-targeting control siRNA), and represent the mean ± SD, n = 5 (B) or 3 (C, D). D) The asterisks indicate statistically significant differences (*p<0.05; **p<0.01; Student's *t* test).

In our initial siRNA screen, several known HCV entry factors, including CD81 and claudin1 were among the host proviral factors [4]. In this study, to identify additional viral entry factors, we performed a targeted siRNA screen of the 205 host factors with both HCVpp and VSV-Gpp, a pseudotyped virus bearing the vesicular stomatitis virus glycoprotein. Silencing of multiple genes inhibited the entry of HCVpp or VSV-Gpp to varying extents (Table S4). Further entry assays with HCVpp of various genotypes and another pseudovirus, the murine leukemia virus pseudoparticle (MLVpp), which uses a clathrin-independent pathway for entry, confirmed the specificity of these genes in HCV entry process (Figures 2A, B).

Five novel HCV-specific entry factors, silencing of which by siRNA significantly restricted the entry of HCVpp but not VSV-Gpp or MLVpp, were identified (Figures 2A, B). They are E-cadherin (CDH1), a major adherens junction protein; the Rho GTPase RAC1; CHKA, a member of the choline kinase family; the SMAD family member SMAD6, a versatile transcriptional modulator of cellular signaling pathways; and CYBA, a subunit of NADPH oxidase involved in cellular oxidative stress response. Three host factors (MAP4, RAB34 and ROCK2) were, for the first time, identified to be required for the entry of both HCV and VSV (Figures 2A, B). Among them, MAP4, a microtubule-associated protein, has been implicated in cellular membrane trafficking and remodeling [26]. Agents disrupting microtubules have been shown to interfere with viral entry including HCV [27], [28]. The reliance of HCV infection on these host factors was confirmed by measuring core protein production and HCV RNA levels in HCVcc-infected cells. Both assays showed siRNAs targeting these entry factors significantly inhibited HCV infection (Figures 2C, D and S2A). Knocking down these HCV-specific entry factors by siRNAs also significantly restricted the infection of HCV of various genotypes and subgenotypes (Figures S2B, C).

**Figure 3:**
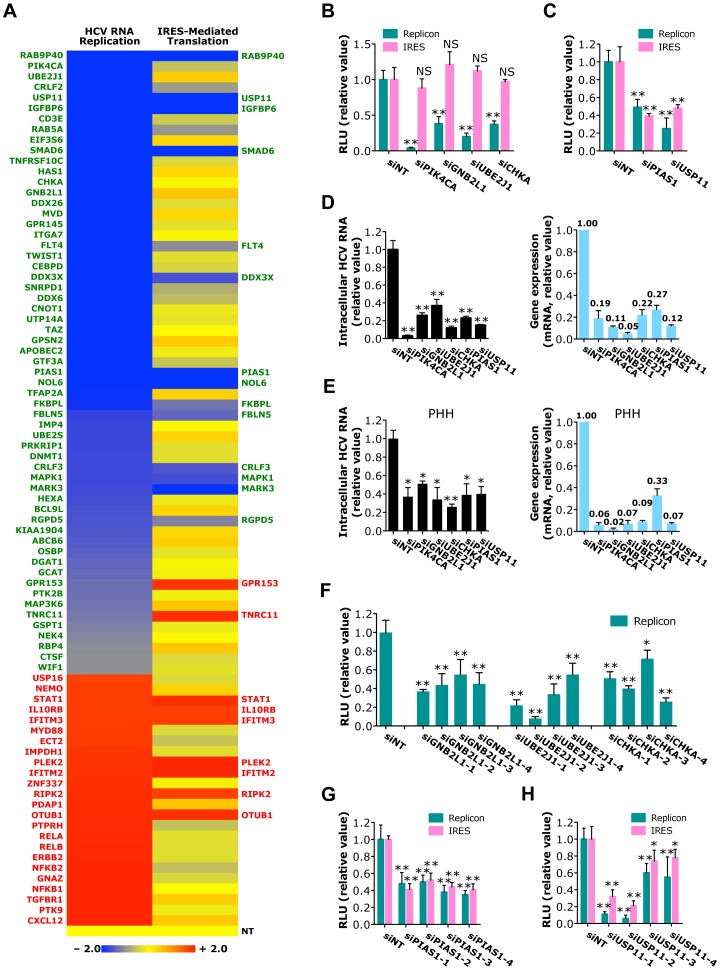
Identification of host factors required for HCV RNA replication or IRES-mediated translation. A) The effects of siRNAs against various host factors on HCV RNA replication (using JFH1-RLuc subgenomic replicon) and IRES-mediated translation (using pHCV-CLX-CMV RNA containing HCV IRES that harbors a firefly luciferase reporter gene). Values relative to siNT in the heatmap are depicted in a continuum of blue (reduced infection - more than 50% decrease of the replicon activity after siRNA-mediated silencing; proviral) to red (enhanced infection - more than 50% increase of the replicon activity after siRNA-mediated silencing; antiviral). Confirmed host factors in each assay are shown in green (proviral) or red (antiviral). B, C) HCV subgenomic replicon or IRES assays of Huh7.5.1 cells depleted of various indicated host factors with siRNAs. Data shown in B and C are subsets of those in Tables S5 and S6. D, E) Quantification of intracellular HCV RNA levels at 48 h post-infection in Huh7.5.1 cells (D) or PHHs (E) pre-treated with various indicated siRNAs. Knockdown efficiencies of various siRNAs in both cell lines were also determined. F) HCV subgenomic replicon assay of Huh7.5.1 cells transfected with GNB2L1, UBE2J1 or CHKA individual siRNAs. G, H) Effects of PIAS1 or USP11 individual siRNAs on HCV RNA replication or IRES-mediated translation, revealed by HCV subgenomic replicon assay or IRES assay, respectively. B–H) All values were normalized to siNT (as 1), and represent the mean ± SD, n = 5 (B, C, F–H) or 3 (D, E). The asterisks indicate statistically significant differences (*p<0.05; **p<0.01); NS, not significant.

Interestingly, PIK4CA, a phosphatidylinositol (PI) 4-kinase important in HCV genome replication [4], [6], [13], [15], [29], [30], was also demonstrated to play a role in mediating viral entry. Depletion of PIK4CA by siRNA in Huh7.5.1 cells significantly blocked HCVpp or VSV-Gpp entry (Figures 2A, B). As expected, silencing of PIK4CA expression in cells also drastically inhibited HCV RNA replication and viral production (Figures 3B, C). PI4KCA hence represents a multifaceted host factor critical for several stages of the HCV life cycle.

### Identification of host factors involved in HCV IRES-mediated translation or RNA replication

SiRNA screen of the 205 genes with HCV subgenomic replicon identified 41 host factors required for viral RNA replication (Figure 3A and Tables S5, S8). Based on their confirmed phenotypes and potential biologic novelty, the most interesting genes are GNB2L1 (RACK1), a subunit of guanine nucleotide binding protein (G protein); UBE2J1, an E2 ubiquitin-conjugating enzyme; and CHKA, a choline kinase that is also involved in HCV entry (Figures 2A, B). Silencing of these genes by siRNAs significantly reduced HCV replication, but had no effect on HCV IRES-mediated translation (Figure 3B), suggesting that these host factors target the RNA replication step of HCV replication cycle.

We further showed that 12 host proteins are specifically involved in HCV IRES-mediated translation (Figure 3A and Tables S6, S8). Genes of particular interest include PIAS1, a member of the mammalian PIAS family that inhibits STAT1-mediated gene activation and DNA binding activity in the nucleus; and USP11, a deubiquitinating enzyme. Silencing of either gene is associated with marked inhibition in both HCV replicon and IRES assays (Figure 3C).

The proviral functions of the above-highlighted host dependencies were observed with the HCVcc system. In both Huh7.5.1 cells and primary human hepatocytes, depletion of these genes by siRNAs significantly inhibited HCV RNA replication and production (Figures 3D, E and S3). The effects of GNB2L1, UBE2J1, CHKA, PIAS1 and USP11 depletion on HCV RNA replication or translation were further characterized by testing their individual siRNAs. Treatment of HCV-infected cells with multiple individual siRNAs significantly reduced HCV replication or translation that are proportional to their knockdown efficiencies (Figures 3F–H and S3).

Families of viral restriction factors that mediate host antiviral immunity were also identified by this approach. Multiple interferon-stimulated genes, components of the NF-κB activation pathway, and novel host factors restricted HCV infection at the RNA replication level, as shown by increased viral replication in cells where expression of these genes was silenced by siRNAs (Figure 3A and Table S5). Similarly, nine host factors were shown to inhibit HCV IRES-mediated translation (Figure 3A and Table S6).

### Identification of host factors associated with HCV assembly or secretion

**Figure 4:**
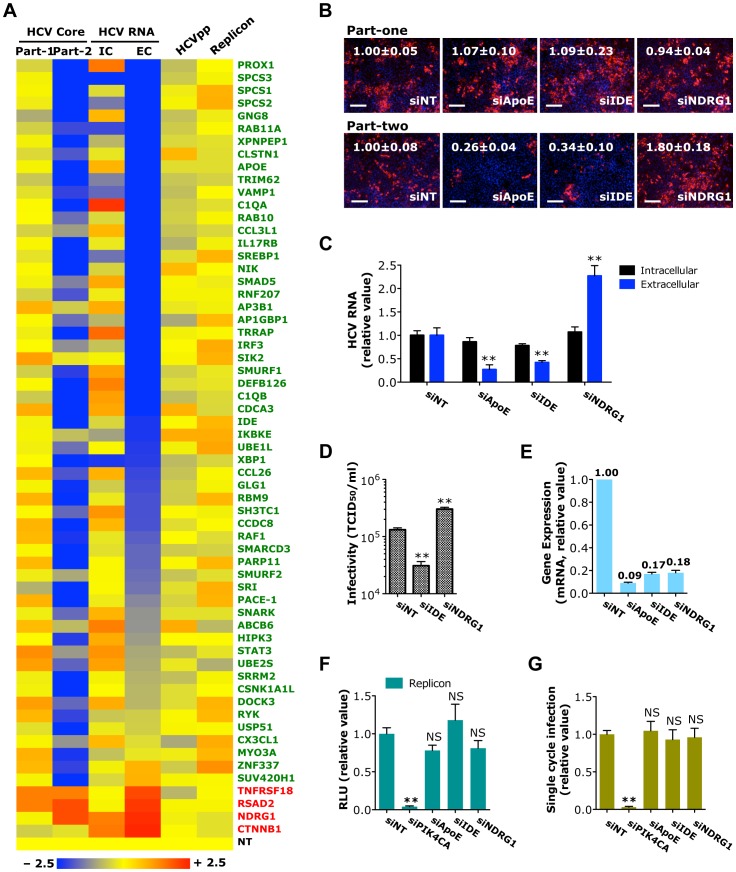
Identification of host factors important in HCV assembly or secretion. A) Effects of siRNA-mediated host factor silencing in HCVcc (core staining and viral RNA quantification), HCVpp and replicon assays shown in a heatmap format. The numerical data are also shown in Tables S2, S3, S4 and S5, respectively. Values relative to siNT in the heatmap are depicted in a continuum of blue (reduced infection) to red (enhanced infection). Gene symbols are displayed on the right. Green represents HPF and red, HAF. Part-1, core staining part-one; Part-2, core staining part-two; IC, intracellular HCV RNA; EC, extracellular HCV RNA. B) Image illustration and quantitative analyses of HCV core staining (part-one and part-two) in Huh7.5.1 cells depleted of indicated host factors. Percentages of core positive cells were quantified, and relative ratios normalized to siNT control (set as 1) were shown. Red: HCV core, blue: nuclei. Magnification 20×. Scale bars represent 100 µm. C, D) Effect of IDE or NDRG1 silencing on HCV RNA production or secretion of infectious HCV (D) in Huh7.5.1 cells. HCV RNA quantification and infectivity assay were performed at 48 h post-infection. E) Knockdown efficiencies of various siRNAs in Huh7.5.1 cells. Gene expression assay was conducted at 72 h after siRNA transfection. F, G) HCV subgenomic replicon assay (F) or quantification of HCVsc infection (G) in Huh7.5.1 cells upon IDE or NDRG1 silencing. SiRNA against PIK4CA or ApoE served as the positive or negative control for both assays, respectively. F, G) Data shown are subsets of those in Tables S5 and S7, respectively. B–G) All values were normalized to siNT (as 1), and represent the mean ± SD, n = 3 (B–E) or 5 (F, G). Asterisks indicate statistically significant differences (**p<0.01); NS, not significant.

The late stages of HCV replication cycle from post-replication to secretion remain largely unexplored. Using a two-part infection protocol with HCVcc system [4] and other virologic assays, we identified 66 host factors that are associated with assembly/secretion (part-two infection) steps of HCV infection (Figure 4A and Tables S2, S3 and S8). The selection criteria were based on comparing effects of siRNA silencing on part-one *vs* part-two infections assay (part-one: no change or minor decrease/increase *vs* part-two: significant decrease/increase), and subsequent HCVpp and HCV replicon assays (no change or minor decrease/increase)(Figure 4A and Tables S2, S3, S4, S5, S6, S7, S8). Genes that clearly fit into these selection criteria include various transcriptional factors/cofactors (TRRAP, SMARCD3, STAT3, IRF3, PROX1, SMAD5, SUV420H1 and ZNF337), kinases and activators of protein phosphorylation (HIPK3, NIK, IKBKE, CSNK1A1L, RYK, RAF1, SNARK and SIK2), small GTPases (RAB11A, ARHGAP22 and GNG8), actin-binding protein (MYO3A), mediator of vesicular transport (VAMP1), protein transporters (RAB10, AP3B1 and AP1GBP1), and those involved in lipid metabolism (SREBP-1, APOE, TRIM62, PLB1 and XBP1). Depletion of these genes by siRNA drastically decreased levels of HCV core staining in part-two of the assay and amounts of HCV RNA secreted into the supernatant, while HCVpp entry or HCV replication/translation was not affected (Figure 4A). In addition, four host antiviral factors (TNFRSF18, RSAD2, NDRG1 and CTNNB1) that specifically restrict HCV infection at the level of assembly or secretion were identified (Figure 4A and Table S8). RSAD2, also known as viperin, is a well-characterized interferon-inducible antiviral factor in HCV infection [31], [32].

Two noteworthy cellular proteins identified in HCV assembly/egress are insulin-degrading enzyme (IDE), a host proviral factor, and N-Myc down regulated Gene 1 (NDRG1), an antiviral host factor. Silencing of IDE by siRNA significantly reduced the percentage of core-positive cells (in part-two only), level of secreted HCV RNA and production of infectious HCV (Figures 4B–E). In contrast, depletion of NDRG1 showed the opposite effects – HCV core and RNA production and viral infectivity were markedly enhanced (Figures 4B–E). SiRNA-mediated knockdown of IDE or NDRG1, however, had no effect on HCV RNA replication and translation or the HCVsc assay (Figures 4F, G), indicating that these genes are predominantly involved in the late stage of HCV replication cycle. We further validated the phenotype-specific roles of IDE and NDRG1 by testing individual siRNAs from the SMART pools and showed that individual siRNAs targeting IDE or NDRG1, like the pools, similarly decreased and enhanced HCV production, respectively (Figure S4).

**Figure 5:**
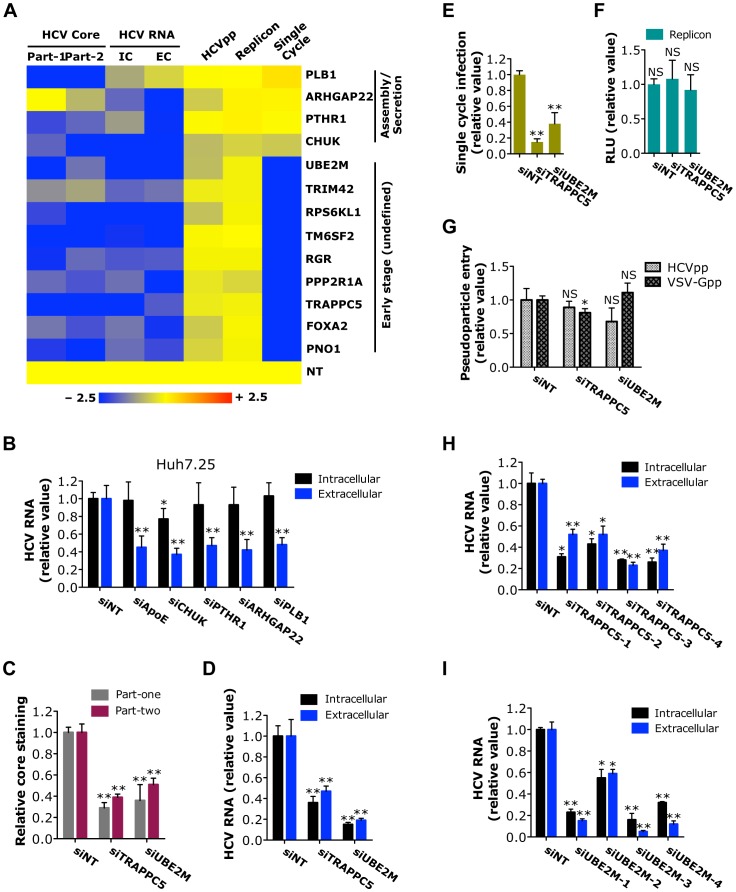
Identification of host factors involved in a yet-to-be-defined early stage of the HCV replication cycle. A) Effects of siRNAs targeting various indicated host factors on HCVcc (core staining and viral RNA quantification), HCVpp, replicon and HCVsc assays are shown in a heatmap. All the numerical data are shown in Tables S2, S3, S4, S5 and S7. Values relative to siNT in the heatmap are depicted in a continuum of blue (inhibition) to yellow (no effect). Gene symbols are displayed on the right. Part-1, core staining part-one; Part-2, core staining part-two; IC, intracellular HCV RNA; EC, extracellular HCV RNA. B) Quantification of intracellular and extracellular HCV RNA levels of CD81-deficient Huh7.25 cells. Cells were treated with various indicated siRNAs for 72 h prior to transfection with full-length JFH-1 HCV RNA. After 48 h, cells and culture media were collected, and viral RNA was extracted and subsequently quantified by Q-RT PCR. C-G) Quantification of HCV core staining part-one and part-two (C), HCV RNA levels (D), HCVsc infection (E), HCV subgenomic replicon RNA (F) and HCVpp and VSV-Gpp entry (G) of Huh7.5.1 cells upon TRAPPC5 or UBE2M silencing. Data shown are subsets of those in Tables S2 (C), S3 (D), S7 (E), S5 (F) and S4 (G), respectively. H, I) Effects of various TRAPPC5 (H) or UBE2M (I) individual siRNAs on HCV infection, determined by measuring intracellular and extracellular HCV RNA levels of Huh7.5.1 cells at 48 h post-infection. B–I) All values were normalized to siNT (as 1), and represent the mean ± SD, n = 3 (B–D, H, I) or 5 (E–G). Asterisks indicate statistically significant differences (*p<0.05; **p<0.01); NS, not significant.

Based on all the virologic assays, a group of genes (*n* = 13) appeared to affect both part-one and part-two of HCVcc infection assay, but had no effect on HCVpp entry or RNA translation and replication (Figure 5A). Single-cycle infection assay was then applied to further define the effects of these genes on HCV life cycle (Table S7). Four host factors (CHUK, PTHR1, ARHGAP22 and PLB1) did not show any effect in the HCVsc assay (Figure 5A). In CD81-deficient Huh7.25 cells that do not support HCV entry but will support a single round of replication after transfection of HCV genomic RNA, siRNAs against these four genes significantly reduced extracellular HCV RNA but not intracellular HCV RNA levels (Figure 5B), thus indicating that these host factors target a late stage of HCV infection - assembly or secretion.

### Host factors involved in undefined HCV post-entry early stage

HCV relies on cellular membrane-trafficking pathways at multiple steps of the viral replication cycle. Particularly, subsequent to viral entry, multiple cellular pathways and mechanisms may be engaged to disassemble and then transport the viral genome to the sites of viral translation and replication. Nine host factors (PNO1, FOXA2, TRAPPC5, PPP2R1A, RGR, TM6SF2, RPS6KL1, TRIM42 and UBE2M) were shown to selectively target an early (undefined) stage of HCV infection based on various virologic assays (Figure 5A). While the HCVpp assay is used routinely to study HCV entry, it is well known that this assay does not completely capture all the entry steps of infectious HCV [33]. For example, NPC1L1, an important entry factor, was not identified through the HCVpp assay [33]. PNO1 is an RNA binding protein localized to the nucleolus; FOXA2, a hepatocyte nuclear factor that serves as a transcriptional activator for liver-specific genes; TRAPPC5, a subunit of the trafficking protein particle complex that has been implicated in vesicular transport [34]; PPP2R1A, a regulatory subunit of protein phosphatase 2 that is implicated in the negative regulation of cell growth and division; RGR, a retinal G-protein coupled receptor; TM6SF2, an integral component of cellular membrane with unknown functions; RPS6KL1, a ribosome protein with kinase activity; TRIM42, a tripartite motif (TRIM) family member with multiple zinc binding domains; and UBE2M, an E2 ubiquitin-conjugating enzyme with versatile activities. None of these factors seem to have obvious functional connections to HCV entry.

Among these host factors required for the undefined post-entry stage of HCV replication cycle, we further validated TRAPPC5 and UBE2M for their phenotypes in HCV infection. Depletion of TRAPPC5 or UBE2M resulted in a significant reduction of HCV infection, in both part-one and part-two HCVcc assays (Figures 5C–E and S5). As expected, HCV entry or replication/translation was not affected by siRNA-mediated knockdown of either gene (Figures 5F, G). Individual siRNAs targeting TRAPPC5 or UBE2M also significantly inhibited HCV RNA production that correlated with the knockdown efficiencies (Figures 5H, I and S5).

## Discussion

**Figure 6:**
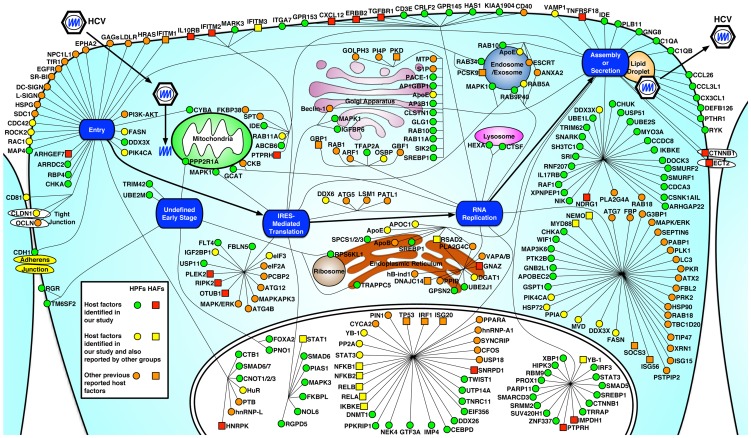
Integrated map of host dependencies in the complete replication cycle of HCV. Using the complete HCV replication cycle (from entry to secretion) as a framework, all verified HCV host dependencies from this study were placed based on their predominant subcellular localization and relevance to particular stages of the viral life cycle. In addition, multiple datasets from other HCV siRNA screens and existing publications were mined, explored and integrated into a comprehensive up-to-date dataset of HCV interacting host factors (see Tables S8 and S9). Computational mapping was performed to reconstitute the map that was further refined manually. HPFs (host proviral factors) are shown in red square, HAFs (host antiviral factors) are shown in green circle. Previously published HCV host dependencies (see Table S9) that were also identified in this study are shown in yellow, and other known HCV host factors that were not identified in this study are shown in orange (HPF in circle and HAF in square).

Understanding the mechanisms of HCV-induced liver disease requires a comprehensive knowledge of how multiple and concurrent pathways are exploited by the virus. Recent systems biology efforts have been successful in discovering novel cellular factors and protein-protein interactions that are important for HCV infection [3], [4], [5], [6], yet many fundamental processes in the complete viral replication cycle remain uncharacterized. In this study, we integrated systems virology and functional genomics approaches and performed extensive functional studies of HCV host dependencies uncovered from our genome-wide siRNA screen with various *in vitro* HCV model systems. We also demonstrated that silencing of these host dependencies does not result in cytotoxicity (Figure S6 and Table S2). In addition, we systematically mined the literature to generate a comprehensive up-to-date database of HCV interacting host factors. As such, a global roadmap of cell regulatory networks and pathways in the entire HCV replication cycle, from entry, viral genome replication and protein translation, to virion assembly and secretion, was constructed (Figures 6, S7 and Table S8). Although there are certainly other HCV host factors that have not yet been defined and this global map will need to be updated in the future, the data set from this study will collectively serve as a valuable resource for further investigation of HCV-host interactions and mechanisms of HCV-related pathogenesis. A comprehensive identification and characterization of HCV host dependencies in the complete viral replication cycle may also reveal potentially valuable targets for prophylactic and therapeutic interventions of HCV infection.

HCV enters host cells through a coordinated cascade of pathways involving multiple entry factors, including CD81 [35], tight junction proteins, CLDN1 [36] and OCLN [37], the receptor tyrosine kinases epidermal growth factor receptor (EGFR) and ephrin receptor A2 (EphA2) [14], and Niemann-Pick C1-like 1 (NPC1L1) [33]. By functional genomics and systematic approaches, we identified previously unappreciated host factors that are specifically required for HCV entry. Among the HCV-specific entry factors identified in this study, RAC1, a Rho GTPase, is an intrinsic component of the macropinocytosis pathway and has been implicated in the entry of a variety of viruses [38]. In HCV infection, RAC1 may mediate actin cytoskeleton rearrangement that allows trafficking of HCV to the TJs [39]. In addition, the role of RAC1 in mediating viral macropinocytosis suggests that HCV may also employ this particular pathway to efficiently infect hepatocytes. CHKA, a member of the choline kinase family involved in the synthesis of phosphatidylcholine [40], likely affects HCV entry by regulating the phospholipid composition of the plasma membrane that has been shown to be important for efficient HCV entry [41]. CYBA is a subunit of NADPH oxidase involved in cellular oxidative stress response and its mechanism in HCV-specific entry requires further experimentation.

Functional studies with HCVpp and HCVcc also identified four shared factors (ROCK2, RAB34, PIK4CA and MAP4) for entry of HCV and VSV, both of which engage a clathrin-mediated endocytosis pathway [42], [43], [44]. ROCK2, a serine/threonine kinase that regulates actin structure reorganization and clathrin transportation [45], probably exerts a proviral function in clathrin-mediated endocytosis of HCV. RAB34, a Golgi-bound small GTPase for protein transport and secretion [46], has been shown to be involved in macropinocytosis and hence viral entry [47]. PIK4CA, a key cellular factor in inducing the formation of the membranous replication complex in HCV-infected cells [15], is also defined as an entry factor for HCV in this study. This observation is consistent with a recent study, in which PIK4CA was shown to be involved in both HCV entry and replication [48]. Upon HCV infection, PIK4CA is recruited by the ER-bound HCV NS5A protein to the membranous web, where its kinase activity is activated by NS5A to produce the membrane lipid PtdIns4P, a mediator of protein attachment [15]. This process is pivotal in facilitating HCV genome replication. The mechanism underlying the role of PIK4CA in mediating HCV entry remains unclear but probably involves membrane re-organization as well.

HCV, like other positive-strand RNA viruses, subverts host membranes to build subcellular vesicles or ER-derived “membranous webs”, in which viral replication occurs [7]. Among the identified host factors associated with viral replication in this study, there is a significant enrichment of factors involved in membrane biogenesis, kinetics, and trafficking; all may be crucial in maintaining the membranous web structure. In addition, various lipid metabolism genes, including multiple lipogenic enzymes were also defined (DGAT1, GPSN2, HEXA, MVD, OSBP and CHKA). These lipid-related genes are co-opted by HCV to create a favorable, lipid-rich microenvironment for viral replication [49]. Other host factors shown to be essential for HCV RNA replication include RNA-binding proteins that facilitate viral RNA synthesis (APOBEC2, DDX26, DDX3X, DDX6, DNMT1, PRKRIP1, and SNRPD1), modulators of protein binding and transport (IMP4, RAB5A, UTP14A and RBP4), kinases and regulators of protein phosphorylation (MAP3K6, NEK4, PIK4CA and PTK2B), and transcriptional factors (CEBPD, GTF3A, TFAP2A, TNRC11, TWIST1 and CNOT1).

Multiple G protein subunits were identified to be important for HCV replication. G proteins like GNB2L1 belong to the larger group of enzymes named GTPases, and play an important role in regulating metabolic enzymes, ion channels, transporters, and transcription. GNB2L1 interacts with HIV-1 Nef and Rev and influenza A virus matrix proteins, and has been implicated in pathogenesis of HIV-1 and influenza A virus infections [50], [51]. Interestingly, recent reports suggested that GNB2L1 as a ribosomal protein regulates eukaryotic translation [52]. In this study, we show that GNB2L1 targets HCV RNA replication only and is not involved in HCV IRES-mediated translation.

Complete cellular pathways and mechanisms involved in HCV assembly and secretion are yet to be delineated. Lipid droplets (LDs) play a crucial role in HCV assembly. In the early step of assembly, the core protein recruits nonstructural proteins and viral replication complexes to LD-associated membranes, but the subsequent steps remain unclear [9]. Multiple host factors have been shown to participate in HCV assembly. Most notable is ApoE, which appears to be incorporated into infectious HCV particles through interaction with NS5A [53], [54]. Other lipid metabolism genes are also required for HCV assembly, for example MTTP, a microsomal triglyceride transfer protein that is essential for VLDL synthesis [55], and DGAT1, a lipogenic enzyme that specifically interacts with and translocates HCV core to the viral assembly sites [56]. We recently demonstrated that DDX3X, CHUK (IKK-α) and SREBP, three host proviral factors identified in our screen, mediate a novel innate pathway to induce lipid droplet formation for viral assembly [57].

The host dependencies for HCV secretion are largely unknown. The current model suggests a role of VLDL secretory pathway in the release of HCV particles [55], [58]. Recently, Coller et al. performed an RNAi screen for host factors involved in HCV egress through live cell imaging of HCV core trafficking in infected cells. Multiple components of the secretory pathway were identified and a trans-Golgi network (TGN) - recycling endosomes - was recently proposed to be involved in HCV secretion [59].

Our functional studies with various HCV *in vitro* systems identified a variety of host factors (including IDE, a proviral factor and NDRG1, an antiviral factor) that are preferentially associated with the assembly/release of HCV. IDE encodes a zinc metallopeptidase that degrades intracellular insulin and thereby terminates insulin activity. Deficiencies in the function of IDE are associated with insulin resistance and type 2 diabetes mellitus, both of which have been associated with chronic HCV infections [60]. IDE is also a cellular receptor that mediates varicella-zoster virus (VZV) entry and cell-to-cell spread [61]. Since HCV has been shown to spread by direct cell-to-cell transfer in cultured hepatocytes [62], we speculate that this function may, at least partially, be mediated by IDE.

NDRG1, a cytoplasmic protein, has been implicated in stress response, cell growth, p53-mediated caspase activation, and iron metabolism [63]. It has also been reported to localize to the endosomes as a RAB4A effector that is involved in vesicular recycling [64]. In addition, NDRG1 was shown to bind to HCV NS5A, though functional implications of this interaction are yet to be defined [3]. Interestingly, in patients infected with HCV, the expression of NDRG1 is significantly down-regulated [65]. We therefore hypothesize that NDRG1 may exert an antiviral effect on HCV assembly/release through its effect on vesicular trafficking in secretion. HCV, on the other hand, may subvert this host antiviral mechanism by down-regulating NDRG1 expression in hepatocytes. In-depth mechanistic studies are needed to further elucidate these important HCV-host interactions.

RNAi-based gene silencing is a powerful approach to systematically study host-virus interactions. Nevertheless, depending on the RNAi platforms and screening assays, both false-positives and negatives present as a barrier to capture all the relevant genes involved in the complete replication cycle of HCV [24], [25]. False-positives can be eliminated by repeated or alternative assays, but false-negatives, of which the selected siRNA/shRNA pools may not silence the respective genes sufficiently, are difficult to assess. However, bioinformatics provide additional tools to identify other factors of importance that may be missed in the screen. Using these approaches to interrogate our data, we create a comprehensive roadmap of interactive networks and pathways for the complete replication cycle of HCV (Figures 6, S7). This map is understandably incomplete and future efforts are necessary to identify additional pathways for its completion. More detailed functional studies of these factors and pathways will likely provide a much more comprehensive and mechanistic understanding of HCV infection.

An important goal of fully characterizing HCV-host interactions is to identify potentially novel host-acting antiviral drugs. The complexity of HCV-host interactions offers a promising venue in therapeutic development. Small molecules that target HCV-dependent host factors, such as cyclosporin-like analogs, are already in clinical development [2]. Identifying novel antiviral molecules may also significantly improve the current landscape of hepatitis C therapy.

## Materials and Methods

### Cell lines and viral propagation and titration

The Huh7 derivative cell lines Huh7.5.1 (provided by Frank Chisari of The Scripps Research Institute, La Jolla, CA) and Huh7.25 (gift from Takanobu Kato and Takaji Wakita of the National Institute of Infectious Disease, Tokyo, Japan) were maintained in complete growth medium (DMEM; Invitrogen) containing 10% FBS (Atlanta Biologicals). Primary human hepatocytes (PHHs) obtained from Steve Strom of the University of Pittsburgh (NIH funded Liver Tissue Procurement and Cell Distribution System) were maintained in Williams E Medium containing cell maintenance supplement reagents (Invitrogen). HCV JFH-1 strain was propagated and infectivity was titrated as previously described [20], [66]. HCV cDNA clones of various genotypes and subtypes (provided by Jens Bukh from Copenhagen University Hospital, Copenhagen, Denmark) used in Figure S2 were JFH-1-based recombinants containing the structural genes (core, E1 and E2), p7, and NS2 of genotype 1a (H77C), 1b (HC-J4), 2b (HC-J8), 3a (S52), 4a (ED43), 5a (SA13), 6a (HK6a), and 7a (QC69), respectively, and viral stocks were prepared and titrated as described previously [67], [68]. HCV infection was conducted at an M.O.I. of 0.5, and assays were typically performed at 48 h post-infection unless otherwise indicated.

### siRNA transfection

siRNAs were transfected into Huh7 cell lines at a 50 nM final concentration, employing a reverse transfection protocol using Oligofectamine (Invitrogen) as previously described [4]. For PHHs, cells were seeded on 12-well plates at 500,000 cells per well and transfected with siRNA at a final concentration of 50 nM using RNAiMAX (Invitrogen). Unless otherwise indicated, further treatments or assays were typically performed 72 h after siRNA transfection, when silencing efficiency reaches maximal.

### HCV core staining part-one (early stage) and part-two (late stage)

Cells were infected with HCV for 48 h, and then fixed with 4% paraformaldehyde (Sigma) for 15 min, permeabilized with 0.3% Triton X-100 (Sigma) in PBS containing 3% FBS (Atlanta Biologicals) and 3% BSA Fraction V (MP Biomedicals) for 30 min, and subsequently immunostained for HCV core protein expression, using purified α-core monoclonal antibody diluted in PBS with 1% BSA. The cells were then incubated with Alexa Fluor 488 or 568 goat α-mouse IgG (Invitrogen) in PBS with 1% BSA, and stained for nuclei with Hoechst 33342 (Invitrogen). Each step was followed by two washes with PBS. Images were captured with an automated Image Express Micro (IXM) microscope (Molecular Devices) and analyzed with the Metamorph Cell Sorting software (Molecular Devices) as previously described [4].

For part-one, Huh7.5.1 cells were treated with various SMARTpool siRNAs for 72 h, and subsequently infected with HCV. After 48 h, cells were immunostained and imaged for HCV core production. This part detects host dependencies involved in the early stages of viral life cycle, from entry to viral protein translation and RNA replication. Culture supernatants of part-one cells were transferred and infected naïve Huh7.5.1 cells, starting part-two, which detects host proteins involved in the later stages of viral infection, including virion assembly and secretion. siRNAs targeting CD81 and ApoE served as proviral controls for part-one and part-two respectively. For both part-one and part-two, the hit selection criteria are: relative values (normalized to siNT as 1) ≤0.5 (Proviral), ≤0.65 and >0.5 (Marginal Proviral), or ≥1.5 (Antiviral), with *P* values<0.05 (see Table S2).

### Viral entry assay

HCV pseudoparticles (HCVpp), VSV-Gpp and MLVpp were generated as previously described [69]. HCVpp harboring E1/E2 glycoproteins from genotypes 1a, 1b, 2a and 4a were derived from the plasmids pHCV7a, pHCV-E1E2.1b3, pCMV-HCV8.1 and pCMV-UKN4 21.16, respectively, as described previously [69], [70]. Huh7.5.1 cells were seeded at the density of 4,500 cells per well in 96-well F-bottom white microplates (Greiner Bio-one), and then transfected with various siRNAs using a reverse transfection protocol. After 72 h, cells were infected with HCVpp of various genotypes, VSV-Gpp, or MLVpp. At 48 h post-infection, cells were lysed in 1× reporter lysis buffer (Promega). Firefly luciferase (Promega) activity was subsequently measured using a POLARstar Omega multidetection microplate reader (BMG Labtech). For the entry assay, the hit selection cutoff points are relative values (normalized to siNT as 1) ≤0.5 (Proviral) or ≥1.5 (Antiviral), with *P* values<0.01 (see Table S4).

### HCV replicon and IRES-mediated translation assays

For replicon assay, Huh7.5.1 cells were treated with various siRNAs for 72 h, and then transfected with JFH1-RLuc subgenomic replicon RNA for 48 h. Cell lysates were then obtained, and *Renilla* luciferase (Promega) activity was determined. For HCV IRES-mediated translation assay, siRNA-treated Huh7.5.1 cells were transfected with pHCV-CLX-CMV RNA (containing HCV IRES) harboring a firefly luciferase reporter gene for 24 h. Firefly luciferase activity was subsequently measured. pHCV-CLX-CMV WT plasmid was provided by Michael Niepmann (Justus-Liebig-University-Giessen, Giessen, German) [18]. For both assays, the hit selection cutoff points are relative values (normalized to siNT as 1) ≤0.5 (Proviral), ≤0.65 and >0.5 (Marginal Proviral) or ≥1.5 (Antiviral), with *P* values<0.05 (see Tables S5 and S6).

### Single cycle HCV infection

Single-round infectious HCV (HCVsc) was generated from a replicon *trans*-packaging system as previously described [23]. Basically, Huh7.5.1 cells were co-transfected with pHH/SGR-Luc plasmid, which carries a bicistronic HCV subgenomic (NS3-5) replicon luciferase reporter with a Pol I promoter/terminator, and a HCV core-NS2 expression plasmid. Culture medium was collected at days 2 to 5 post-transfection containing HCV-like infectious particle (HCV-LP) [23] which is deficient of core protein. The core assembly-defective HCV-LP can efficiently replicate viral RNA in cells but is unable to produce progeny virus, thereby permitting experimental separation of HCV genome replication and virion assembly as two distinct steps [23]. We named this single-round infectious HCV as HCVsc. In the single cycle infection assay, Huh7.5.1 cells were first treated with various siRNAs for 72 h before infection with HCVsc. At 48 h post-infection, cells were harvested and firefly luciferase activity was measured. The hit selection cutoff points are relative values (normalized to siNT as 1) ≤0.5 (Proviral) with *P* values<0.05 (see Table S7).

### Viral infectious titer assay

HCV titration was performed in 96-well clear bottom black assay plates (Corning) seeded with 1×10^4^ Huh7.5.1 cells per well. Viral supernatants were serially diluted by 10-fold in complete growth medium and used to infect the seeded cells (in 8 replicates). At 48 h post-infection, cells were immunostained for HCV core protein as described above. Wells that contain at least one core-expressing cell were counted as positive, and the TCID_50_ was calculated [19].

### Viral RNA isolation and quantification

Total RNA was isolated with RNeasy Mini Kit (Qiagen) from cells, or with QIAamp Viral RNA Mini Kit (Qiagen) from supernatants. Copy numbers of intracellular and extracellular HCV RNA were determined by quantitative RT-PCR with the probe, primers, and parameters described previously [4]. The relative amount of intracellular HCV RNA was normalized to the internal control human 18S rRNA (Applied Biosystems). For the siRNA screen, the hit selection cutoff points are relative values (normalized to siNT as 1) ≤0.5 (Proviral), ≤0.65 and >0.5 (Marginal Proviral), or ≥1.5 (Antiviral), with P values<0.05. Relative values <1.5 with *P* values≥0.05 are regarded as “Marginal Antiviral” factors (see Table S3).

### *In Vitro* transcription of HCV RNA and transfection

HCV subgenomic replion RNAs were linearized with Xba I and purified by phenol-chloroform-isoamyl alcohol extraction. *In vitro* transcription was performed by using the MEGAscript T7 kit (Ambion), according to the manufacturer's protocol. The quality and quantity of RNA were evaluated by NanoDrop spectrophotometer (Thermo Scientific). Aliquots (22 µl, 1 µg/µl) of RNA were stored frozen at −80°C until use. RNA transfection was performed using DMRIE-C Reagent (Invitrogen) according to the manufacturer's instructions.

### Gene expression assay

Total cellular RNA was prepared with RNeasy Mini Kit (Qiagen). Complementary DNA (cDNA) was synthesized from total cellular RNA with First Strand cDNA Synthesis Kit (Roche). The mRNA levels of target genes were quantified by quantitative PCR using gene-specific primers and probes (IDT) and TaqMan Gene Express Master Mix (Applied Biosystems) on an ABI 7500 Real Time PCR System. Relative mRNA levels were calculated using the ΔΔ*CT* method, with 18S rRNA as the internal control for normalization.

### ATPlite assay

Huh7.5.1 cells (10,000 cells per well) cultured in 96-well white assay plates (Greiner Bio-One) were treated with various siRNAs at a final concentration of 50 nM each for 72 h. Cells were then harvested and lysed with 50 µL of mammalian cell lysis solution (PerkinElmer). After 5 min, 50 µL of ATPlite substrate solution (PerkinElmer) was added, and the luminescence ATPlite activity in each well was measured using a POLARstar Omega multidetection microplate reader.

### Statistical analysis

Results are presented as the means ± s.d. The two-tailed unpaired Student's *t* test was used for statistical analysis. The level of significance is denoted in each figure (* *P*<0.05; ** *P*<0.01; NS, not significant).

## Supporting Information

Figure S1 **Schemes of the functional genomics approaches for global identification/characterization of HCV host dependencies.** As a follow-up of the primary genome-wide siRNA screen and subsequent bioinformatics meta-analyses [4], 205 host factors were selected based on their functional information and potential relevance to the HCV life cycle. These genes were subjected to various virologic assays as demonstrated through functional genomics approaches. A detailed evaluation of the data and reconciliation of discrepancies designated these host factors to various steps of HCV life cycle. HPF: host proviral factors; HAF: host antiviral factors.
(TIF)

Figure S2 **Effects of siRNAs targeting various indicated viral entry factors on HCVcc (of multiple genotypes) infection.** A) Knockdown efficiencies of siRNAs targeting various indicated host factors in Huh7.5.1 cells. Gene expression assay was performed at 72 h after siRNA transfection. B, C) Quantification of intracellular HCV RNA levels (B) and siRNA-mediated knockdown efficiencies (C) in Huh7.5.1 cells pre-treated with various indicated siRNAs and subsequently infected with HCV of multiple genotypes. HCV infection was allowed for 48 h. All values were normalized to siNT control (as 1), and represent the mean ± SD, n = 3.
(TIF)

Figure S3 **SiRNA-mediated knockdown of various host factors associated with HCV RNA replication or IRES-mediated translation.** Huh7.5.1 cells were transfected with various individual siRNAs of GNB2L1 (A), UBE2J1 (B), CHKA (C), PIAS1 (D) or USP11 (E) for 72 h, and then infected with HCV. Intracellular HCV RNA levels and siRNA-mediated knockdown efficiencies were measured at 48 h post-infection. Values are shown relative to siNT (as 1), and represent the mean ± SD, n = 3 throughout. Asterisks indicate statistically significant differences (*p<0.05; **p<0.01).
(TIF)

Figure S4 **Phenotype-specific roles of IDE and NDRG1 in modulating HCV assembly/secretion.** Huh7.5.1 cells were treated with various individual siRNAs against IDE (A) or NDRG1 (B) for 72 h, and then infected with HCV. Potencies of individual siRNAs in restricting productive HCV infection and inhibiting relevant gene expression were determined by measuring intracellular and extracellular HCV RNA levels and IDE or NDRG1 mRNA levels. Values are shown relative to siNT (as 1), and represent the mean ± SD, n = 3 throughout. Asterisks indicate statistically significant differences (*p<0.05; **p<0.01).
(TIF)

Figure S5 **Potencies of SMARTpool or individual TRAPPC5 (A) or UBE2M (B) siRNAs in silencing relevant gene expression in Huh7.5.1 cells.** Gene expression assay was performed at 72 h after siRNA transfection by Q-RT PCR. All values were normalized to siNT (as 1), and represent the mean ± SD, n = 3 throughout.
(TIF)

Figure S6 **Test of cytotoxicity of siRNAs used in this study.** Huh7.5.1 cells were treated with various indicated siRNAs at the final concentration of 50 nM each for 72 h, ATPlite activities that represent proliferation and cytotoxicity of cultured cells were subsequently quantified. Values (in relative luciferase unit, RLU) were normalized to siNT (as 1), and represent the mean ± SD, n = 5.
(TIF)

Figure S7 **Integrated map of cellular pathways in the HCV replication cycle.** Using the complete data set from Table S1, S2, S3, S4, S5, S6, S7, S8, S9, statistically significant cellular pathway maps (based on enrichment distribution sorting) and Gene Ontology (GO) cellular and molecular functions that are associated with HCV replication cycle are shown. The network is shown schematically in an HCV-infected hepatocyte as the background. Red: HCV host factors characterized in this study (red). White: bridging proteins that were not identified but are functionally relevant.
(TIF)

Table S1 **Host factors investigated in the study.**
(XLS)

Table S2 **HCVcc assay_core staining, cell viability.**
(XLS)

Table S3 **HCVcc assay_viral RNA quantification.**
(XLS)

Table S4 **Viral entry assays.**
(XLS)

Table S5 **HCV subgenomic replicon assay.**
(XLS)

Table S6 **HCV IRES-mediated translation assay.**
(XLS)

Table S7 **HCV single cycle infection assay.**
(XLS)

Table S8 **Summary_all life cycle stages.**
(XLS)

Table S9 **HCV host factors – reported.**
(XLS)
