# Supplementary figures and images for: Integrative Functional Genomics of Hepatitis C Virus Infection Identifies Host Dependencies in Complete Viral Replication Cycle
Source: PLoS Pathog. 2014 May 22;10(5):e1004163. doi: 10.1371/journal.ppat.1004163 (PMC4095987; doi:10.1371/journal.ppat.1004163)

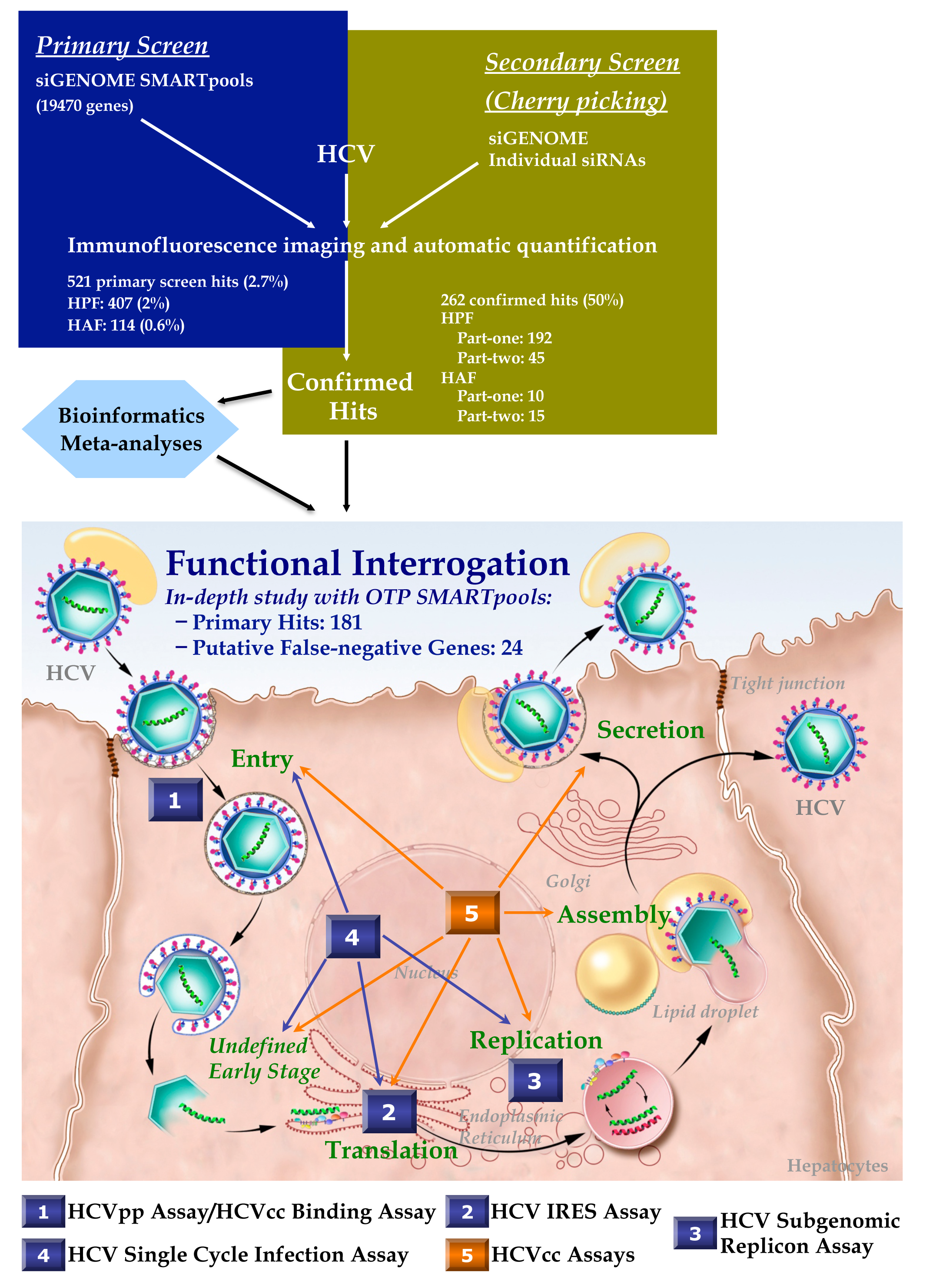

Supplement: Figure S1 — Schemes of the functional genomics approaches for global identification/characterization of HCV host dependencies. As a follow-up of the primary genome-wide siRNA screen and subsequent bioinformatics meta-analyses [4], 205 host factors were selected based on their functional information and potential relevance to the HCV life cycle. These genes were subjected to various virologic assays as demonstrated through functional genomics approaches. A detailed evaluation of the data and reconciliation of discrepancies designated these host factors to various steps of HCV life cycle. HPF: host proviral factors; HAF: host antiviral factors. (TIF) [file ppat.1004163.s001.tif]

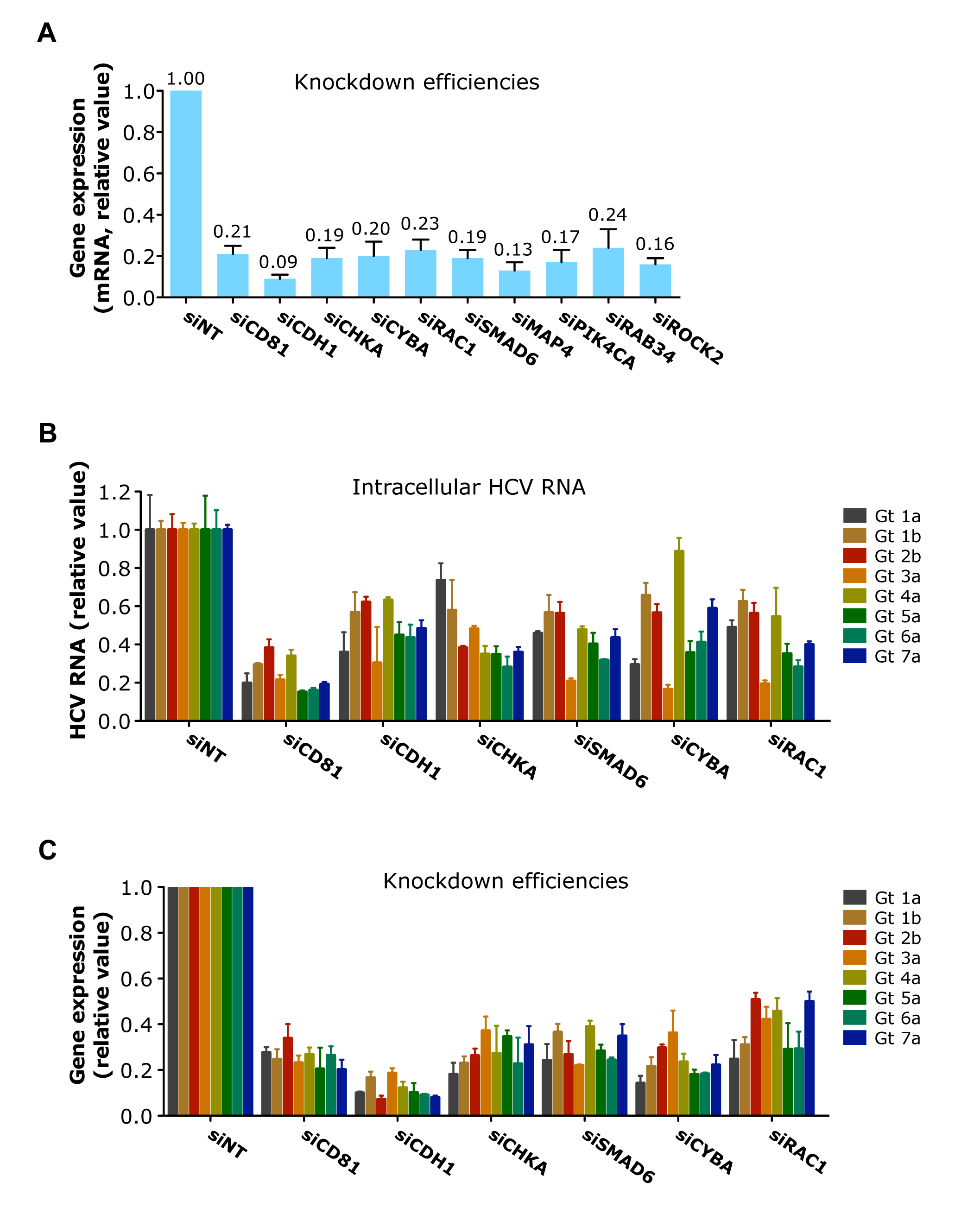

Supplement: Figure S2 — Effects of siRNAs targeting various indicated viral entry factors on HCVcc (of multiple genotypes) infection. A) Knockdown efficiencies of siRNAs targeting various indicated host factors in Huh7.5.1 cells. Gene expression assay was performed at 72 h after siRNA transfection. B, C) Quantification of intracellular HCV RNA levels (B) and siRNA-mediated knockdown efficiencies (C) in Huh7.5.1 cells pre-treated with various indicated siRNAs and subsequently infected with HCV of multiple genotypes. HCV infection was allowed for 48 h. All values were normalized to siNT control (as 1), and represent the mean ± SD, n = 3. (TIF) [file ppat.1004163.s002.tif]

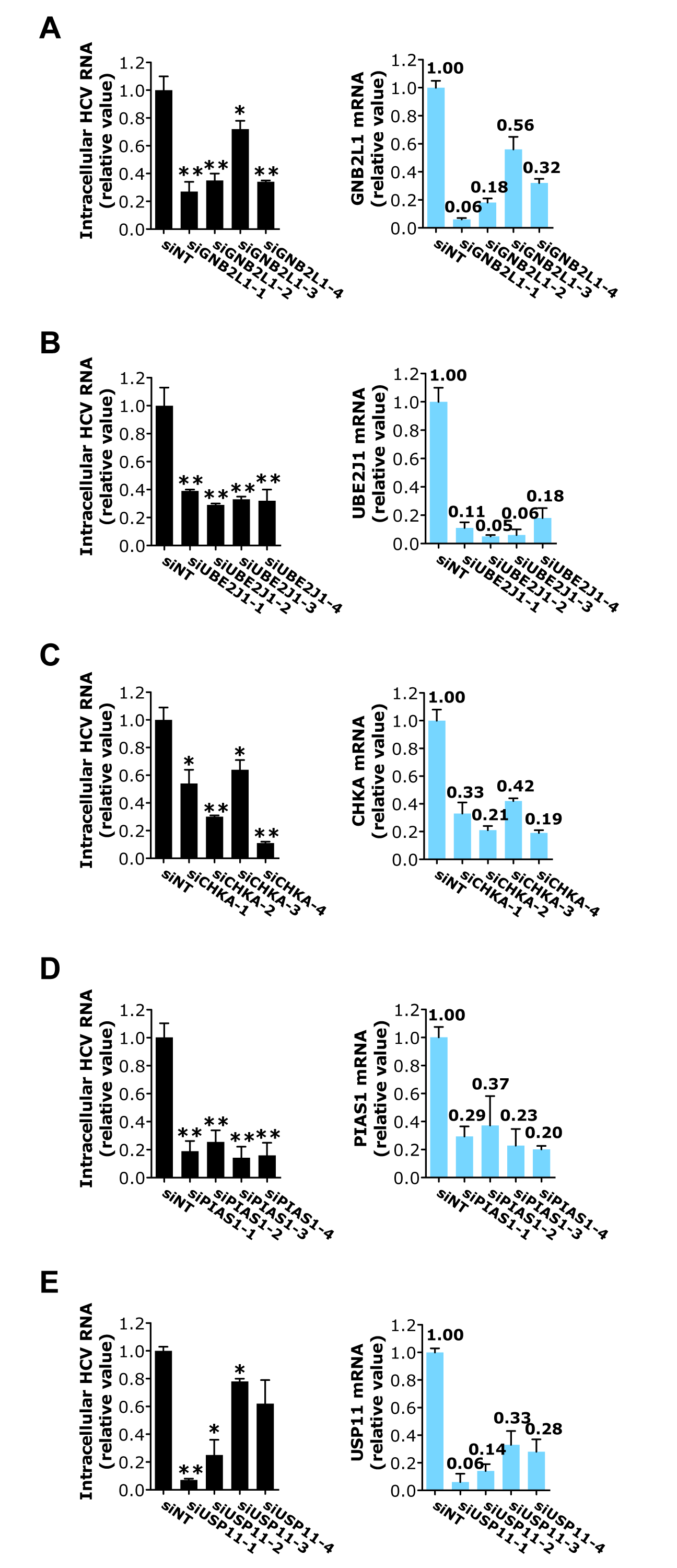

Supplement: Figure S3 — SiRNA-mediated knockdown of various host factors associated with HCV RNA replication or IRES-mediated translation. Huh7.5.1 cells were transfected with various individual siRNAs of GNB2L1 (A), UBE2J1 (B), CHKA (C), PIAS1 (D) or USP11 (E) for 72 h, and then infected with HCV. Intracellular HCV RNA levels and siRNA-mediated knockdown efficiencies were measured at 48 h post-infection. Values are shown relative to siNT (as 1), and represent the mean ± SD, n = 3 throughout. Asterisks indicate statistically significant differences (*p<0.05; **p<0.01). (TIF) [file ppat.1004163.s003.tif]

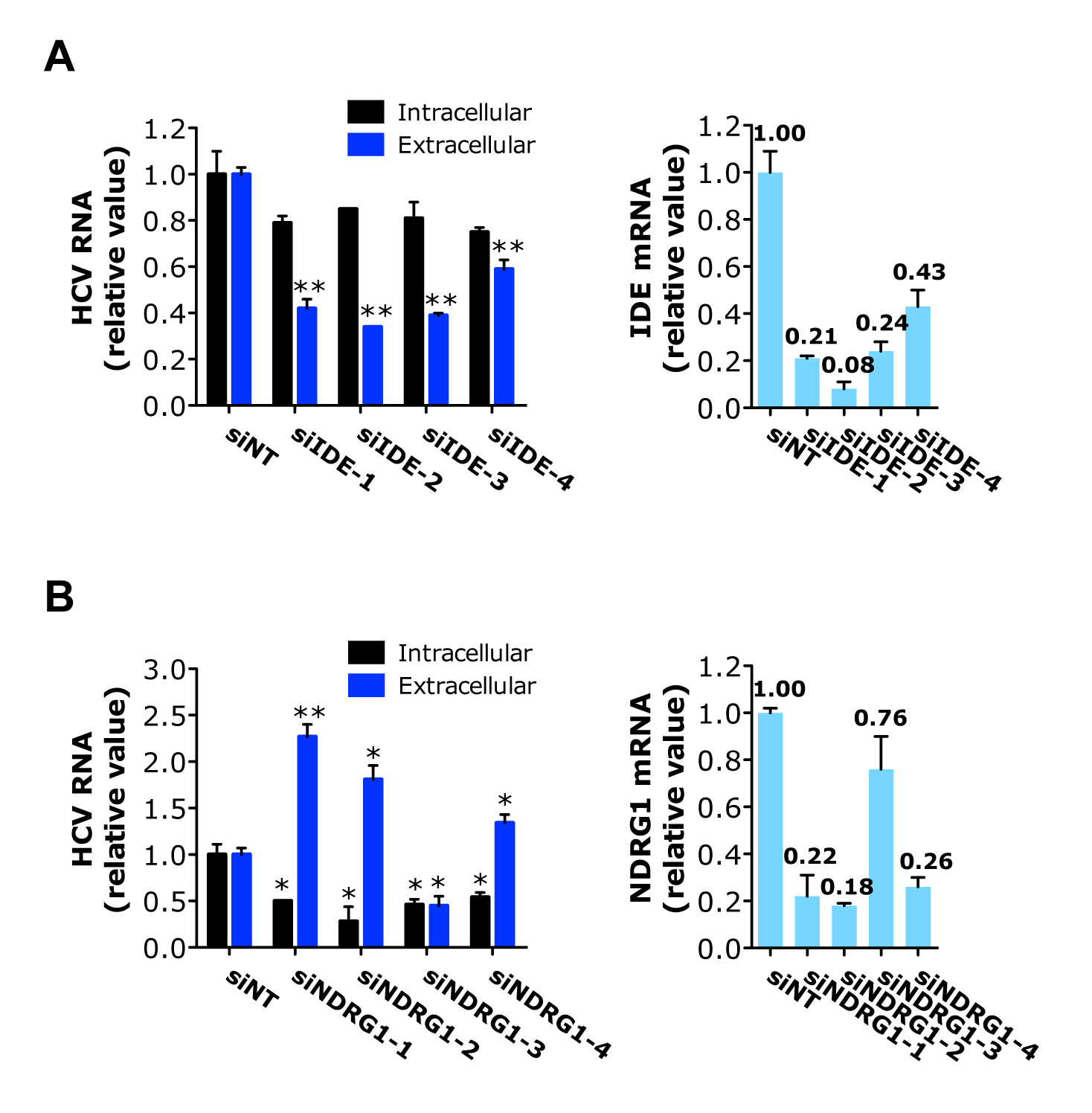

Supplement: Figure S4 — Phenotype-specific roles of IDE and NDRG1 in modulating HCV assembly/secretion. Huh7.5.1 cells were treated with various individual siRNAs against IDE (A) or NDRG1 (B) for 72 h, and then infected with HCV. Potencies of individual siRNAs in restricting productive HCV infection and inhibiting relevant gene expression were determined by measuring intracellular and extracellular HCV RNA levels and IDE or NDRG1 mRNA levels. Values are shown relative to siNT (as 1), and represent the mean ± SD, n = 3 throughout. Asterisks indicate statistically significant differences (*p<0.05; **p<0.01). (TIF) [file ppat.1004163.s004.tif]

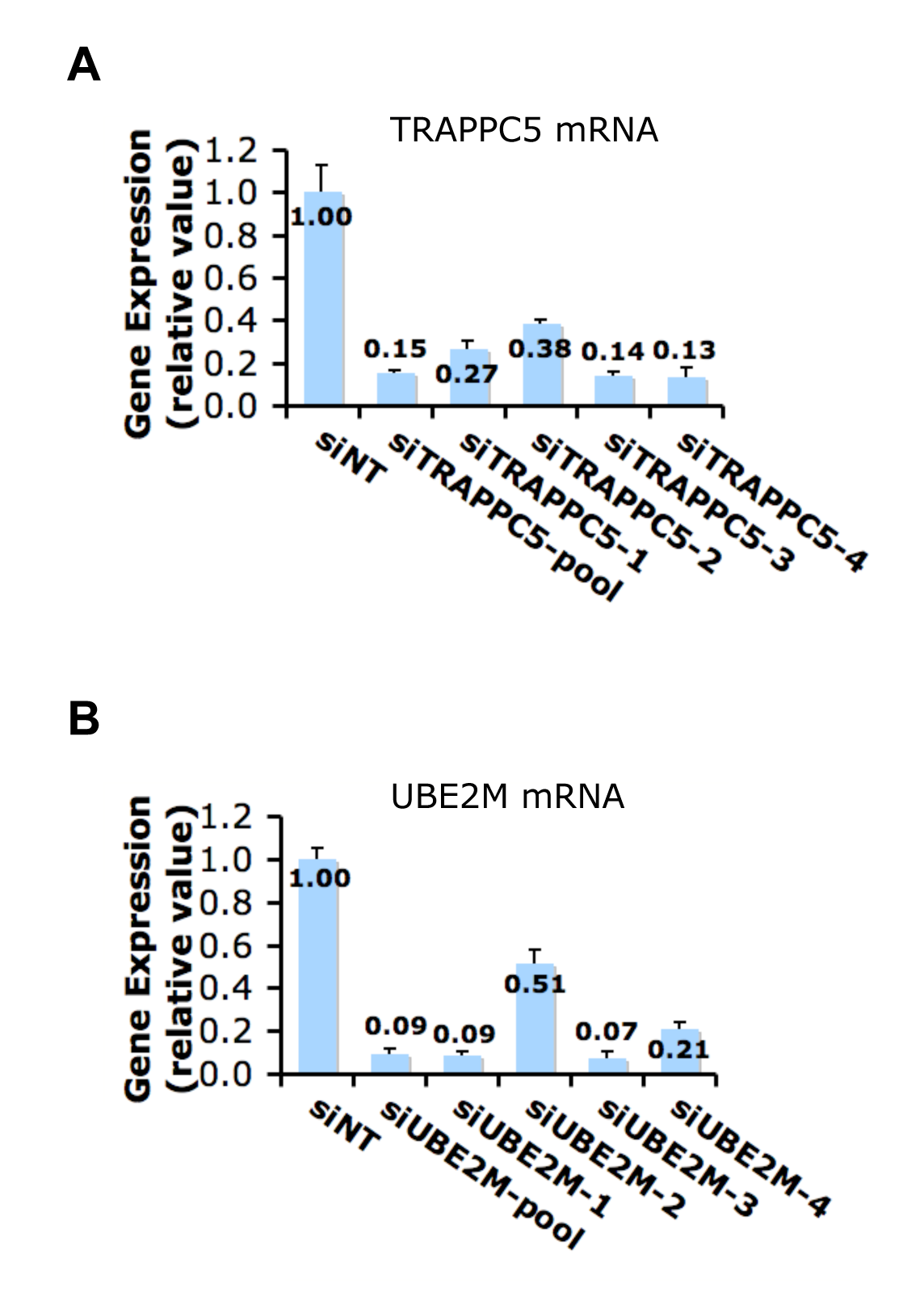

Supplement: Figure S5 — Potencies of SMARTpool or individual TRAPPC5 (A) or UBE2M (B) siRNAs in silencing relevant gene expression in Huh7.5.1 cells. Gene expression assay was performed at 72 h after siRNA transfection by Q-RT PCR. All values were normalized to siNT (as 1), and represent the mean ± SD, n = 3 throughout. (TIF) [file ppat.1004163.s005.tif]

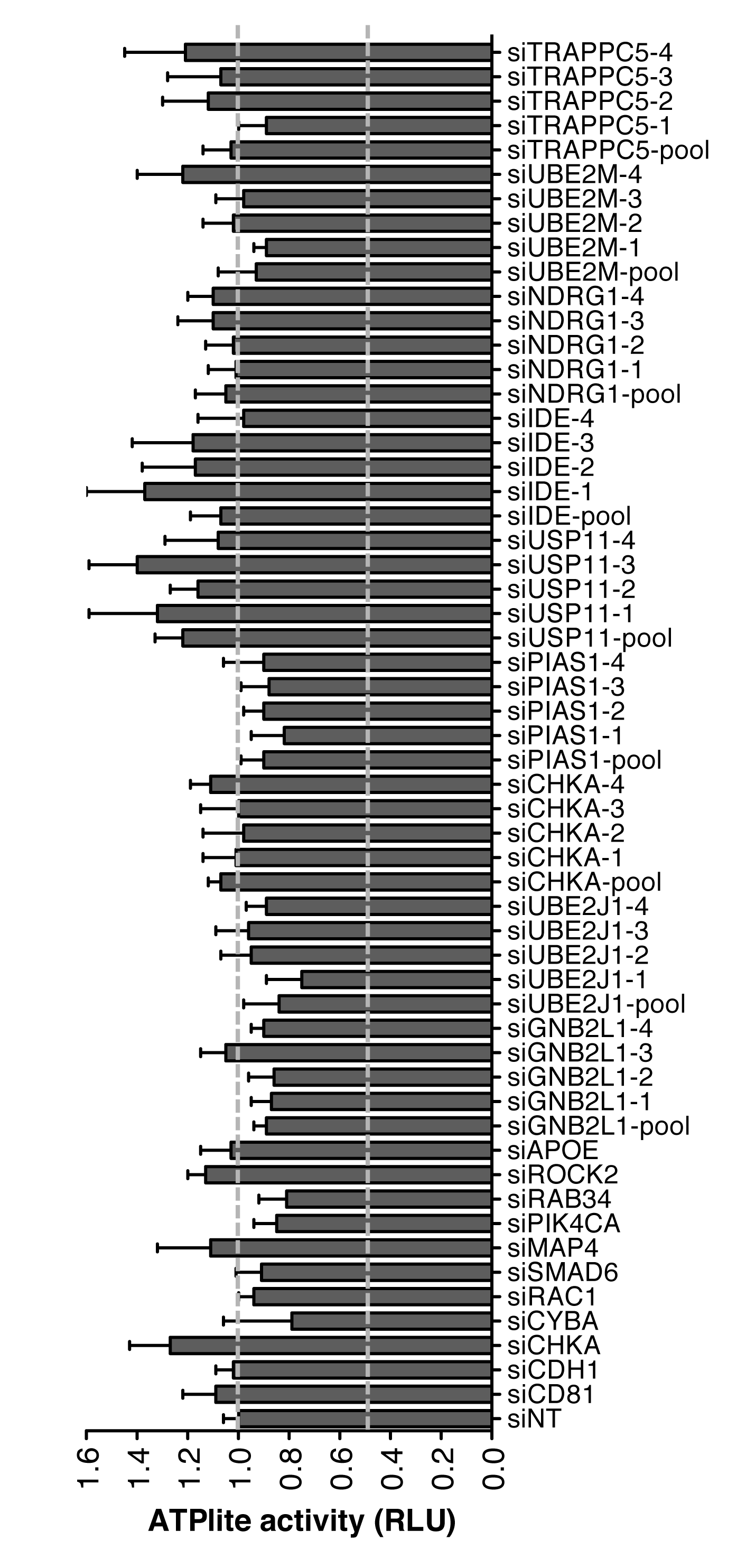

Supplement: Figure S6 — Test of cytotoxicity of siRNAs used in this study. Huh7.5.1 cells were treated with various indicated siRNAs at the final concentration of 50 nM each for 72 h, ATPlite activities that represent proliferation and cytotoxicity of cultured cells were subsequently quantified. Values (in relative luciferase unit, RLU) were normalized to siNT (as 1), and represent the mean ± SD, n = 5. (TIF) [file ppat.1004163.s006.tif]

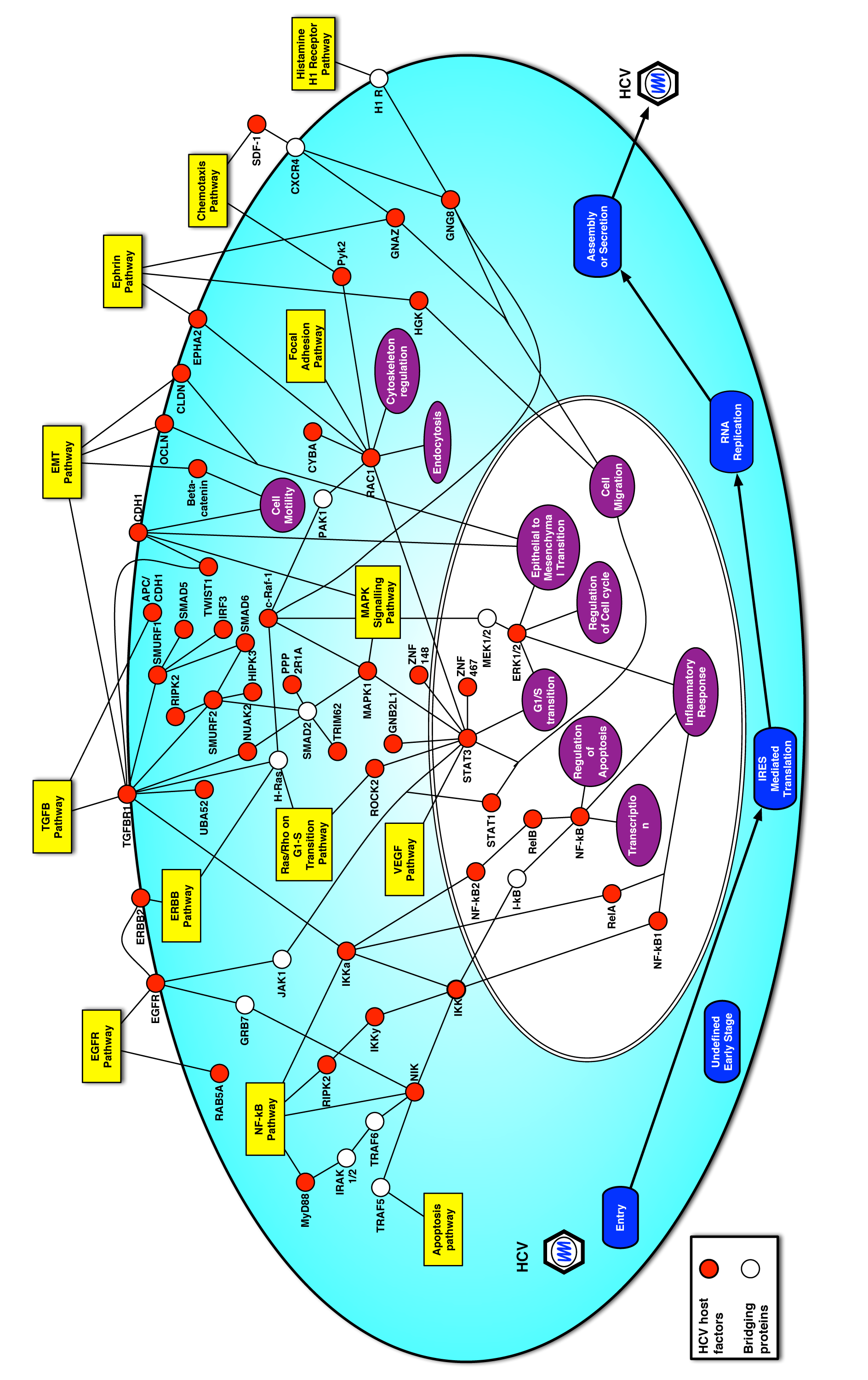

Supplement: Figure S7 — Integrated map of cellular pathways in the HCV replication cycle. Using the complete data set from Table S1, S2, S3, S4, S5, S6, S7, S8, S9, statistically significant cellular pathway maps (based on enrichment distribution sorting) and Gene Ontology (GO) cellular and molecular functions that are associated with HCV replication cycle are shown. The network is shown schematically in an HCV-infected hepatocyte as the background. Red: HCV host factors characterized in this study (red). White: bridging proteins that were not identified but are functionally relevant. (TIF) [file ppat.1004163.s007.tif]
